# Do Consumers Value Agri-Food Industries’ Environmental Commitment? The Case of the Table Olive Industry

**DOI:** 10.3390/foods13132131

**Published:** 2024-07-04

**Authors:** Patricia Rus-Fernández, Alba Sánchez-Torres, Isabel Fernández-Segovia, Ana Fuentes

**Affiliations:** University Institute of Food Engineering-FoodUPV, Universitat Politècnica de València, 46022 Valencia, Spain; patrufer@upv.es (P.R.-F.); albasancheztorres5196@gmail.com (A.S.-T.); isferse1@tal.upv.es (I.F.-S.)

**Keywords:** consumer behaviour, sustainability, consumer attitude, environmental commitments, table olive industry

## Abstract

An increasing number of food companies are voluntarily adopting environmental policies and sustainability initiatives to tackle climate change. The aims of this study were to analyse the presence of environmental labels on table olive products, to explore consumer perceptions of these companies’ environmental commitment and initiatives, and to evaluate the influence of these messages on purchasing decisions. For this purpose, a market study was conducted in different hypermarkets and supermarkets in Spain, and an online survey was submitted to consumers (*n* = 227). The results show that environmental claims and/or certifications related to sustainability do not appear on table olive products, despite most of the companies that produce and/or market table olives having adopted environmental and sustainability policies and commitments (34.3% have their environmental policy published on their website). More than 85% of consumers positively value these companies’ sustainability commitments and consider environmental initiatives to be very important. As a sector of consumers pays close attention to environmental commitments, it would be interesting for table olive companies to identify their sustainability policies on their products’ labelling to, thus, facilitate pro-environmental consumer purchase choices. These results could help the food industry develop the best strategies to publicise their social and environmental policies and commitments.

## 1. Introduction

Sustainability is one of the main objectives of the European market, in addition to other new trends like digitalisation, health awareness, and the promotion of vegetable alternatives [1]. To achieve these objectives, the European Union (EU) seeks to become a sustainable and climate-neutral region by 2050 through its European Green Deal policies. It established mandatory legislation in 2021 to ensure that companies took part in the proposed commitments (2020/2129 (INL)). In line with this strategy, the Sustainable Development Goals (SDGs) proposed by the United Nations (UN) are relevant to the global economy and necessary for a greener planet [2]. To fulfil these goals, companies are starting to implement carbon-neutral strategies for their brands and products [3].

According to the International Olive Council (IOC), the world’s table olive production for the 2022/2023 season amounted to approximately 3 million tonnes. The EU contributed 27% of the total production. The EU’s largest producers are Spain, Greece, and Italy (50%, 39%, and 7% of the total EU production, respectively) [4]. The table olive sector has an environmental impact along its production chain, from the cultivation, food processing, and distribution phases to the end-of-life phase. Recently, table olive companies have made important environmental commitments, which are reflected in their corporate environmental policies. To date, companies have based their environmental actions on the adoption of good practices and implementing voluntary Environmental Management Systems based on the ISO 14001 standard or the EU’s Eco-Management and Audit Scheme (EMAS) [5]. The ISO 14001 standard is the main reference model related to environmental management that helps organisations minimise the negative impact of their operations on the environment and comply with the applicable laws and regulations. The requirements of ISO 14001 are an integral part of the EMAS system, which is considered more demanding mainly concerning performance improvement, legal compliance, and reporting duties. In addition to these voluntary certifications, companies are demonstrating their environmental commitment by other initiatives, such as employing biodegradable or reusable packaging and renewable energies, or with new certifications or sustainability seals. Indeed, some companies are creating product labels with actual environmental impacts, but these labels are usually too specific and not shared by the rest of their products [6]. By using these labels, companies attempt to promote consumer purchasing, but do not always have the intended effect on consumers. On the contrary, different studies have established that a segment of consumers considers the price of products with sustainable labels to be relatively high compared to the product’s quality [7,8]. Although no country has introduced an environmental label based on environmental impact, recent research has shown that an easy-to-understand labelling method common to all food products would help consumers to choose more sustainable products [9].

Consumer concerns about the food system have increased, as reflected by consumer demand for attributes that suit their social and ethical priorities, along with growing pro-environment consumerism. Given the growing concern about the environment, recent studies suggest that companies should provide consumers with information about their environmental commitment, which could be reflected in consumer purchases [10,11]. Nonetheless, some studies have pointed out that consumers perceive a disconnection between what companies communicate and their corporative environmental performance [12]. Indeed, do Paço and Reis [13] established that scepticism about environmental claims is particularly noticeable in consumers who are very environmentally aware. Concerning this fact, knowing how consumers value companies’ sustainability policies and checking how consumer scepticism about sustainability commitments could affect their purchase behaviours would be very interesting topics of study for the food industry. Therefore, table olive manufacturers should know the impact of such information on consumers to design better strategies that improve sustainability while increasing their competitiveness on the market.

In light of these considerations, this research work aimed to analyse the presence of environmental labels on the table olive products currently on the Spanish market, explore consumer perceptions of these companies’ environmental commitment, and analyse the influence of these messages on consumers’ purchasing decisions.

## 2. Methods

### 2.1. Market Research

A market study was conducted in different hypermarkets and supermarkets in Spain to analyse the supply of table olive products available in physical stores and via online sales. The objective of this part of the study was to identify the nutritional and environmental claims on table olive products. For this purpose, the collected data were the following: brand name, olive variety, preparation and ingredients, sale formats, nutritional claims, and environmental claims and/or certifications. The websites of the leading companies in the Spanish table olive sector were also visited to check their environmental commitments and policies and their environmental and/or social responsibility certifications.

### 2.2. Consumer Perception Survey

An online questionnaire was created using the Google Surveys platform to evaluate consumer perceptions of table olive companies’ environmental commitment and to assess the importance that consumers attach to these aspects for their purchasing decisions. Research was conducted in compliance with regulations related to studies with human participants, as established by the Research Ethics Committee of the Universitat Politècnica de València, and in compliance with the Declaration of Helsinki. On the first survey page, participants had to tick a box to confirm that they had read the information form, they met the inclusion criteria, and they accepted the conditions of the study. Participants were assured that their responses would remain confidential and would be fully anonymised so they could not be traced back to them.

Respondents were recruited through word-of-mouth and social networks. With a sample size of 227 valid cases and by assuming a 95% confidence level, the sampling error of the present study was 6.5%.

The questionnaire had been previously pretested with 20 consumers. It comprised three parts, i.e., socio-demographic data collection, consumer opinions of table olives companies’ environmental commitment, and checking packaging information.

#### 2.2.1. Socio-Demographic Data

In the first questionnaire part, the socio-demographic data from participants were collected and included gender, age, level of education, monthly family income, and the province they live in.

#### 2.2.2. Consumer Opinion of Table Olives Companies’ Environmental Commitment

**Figure 1:**
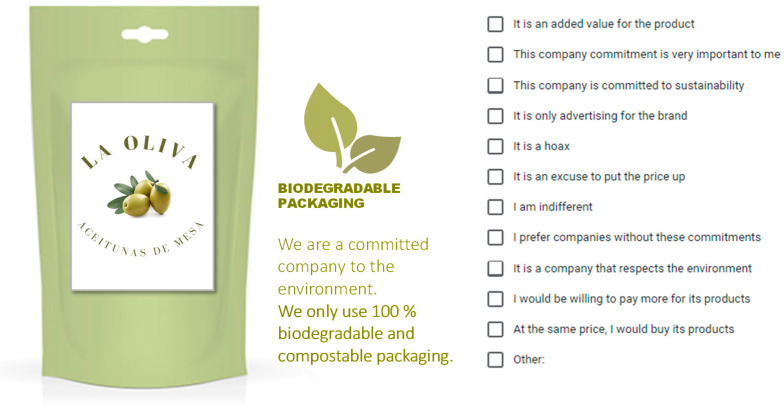
Example of the images presented in the questionnaire.

In this part, a CATA (check-all-that-apply) analysis was performed. Participants were presented with five images of table olive packaging, each showing different messages related to food companies’ environmental policies. These environmental and sustainability commitments were related to the following: “using renewable energies”, “reducing CO_2_ emissions”, “lowering the water footprint”, “zero waste”, “employing biodegradable packaging”, and “organic production”. An example of the images presented to respondents is shown in Figure 1.

Respondents were asked what each commitment suggested to them. To answer this question, respondents had 11 closed options and a final “other” option, in which there was space to indicate any other assessment that was not covered by the provided indications. Of these options, a similar number of negative (4, 5, 6, 7, 8) and positive (1, 2, 3, 9, 10, 11) statements was given. Respondents could mark all the statements with which they agreed. Options were as follows:

(1) It is an added value for the product.
(2) This company commitment is very important to me.
(3) This company is committed to sustainability.
(4) It is only advertising for the brand.
(5) It is a hoax.
(6) It is an excuse to put the price up.
(7) I am indifferent.
(8) I prefer companies without these commitments.
(9) It is a company that respects the environment.
(10) I would be willing to pay more for its products.
(11) At the same price, I would buy its products.
(12) Other: __________________________________.

Respondents were also asked if they consult the social and environmental commitments of the companies that manufacture the products they usually buy. The answer options were as follows:

Yes, I only buy products made by companies committed to the environment.

Yes, I usually consult companies’ social and environmental commitments.

No. Although I’m interested in knowing which companies are more committed, I do not look for this information.

No. Although I’m interested, I do not know how to obtain this information.

No, because I think that most of it is advertising and I don’t find it useful.

Other: ________________________________.

#### 2.2.3. Checking Packaging Information

Finally, the questionnaire ended with several questions related to the frequency with which respondents consulted the environmental, nutritional, and organic production and animal welfare information that may appear on product labels. A 5-point Likert scale was used, where 1 corresponded to “Never” and 5 to “Always”.

### 2.3. Statistical Analysis

During market research, a correspondence analysis was carried out to visualise any possible relations between companies’ sustainability commitments and their size.

In the CATA analysis, the frequency of each option was determined by counting the number of respondents who selected that option. Cochran’s Q test was employed to evaluate if there were significant differences in the frequency of options among environmental commitments. Two correspondence analyses were also carried out to visualise the relations between statements and environmental commitments on a two-dimensional map.

A Kruskal–Wallis analysis was carried out to determine if there were significant differences in the frequency with which consumers check nutritional, sustainability, and animal welfare information. Dunn’s test with Bonferroni correction was used to test for differences at the 5% significance level. A hierarchical cluster analysis, using Ward’s agglomeration method and Euclidean distance, was performed to categorise similar respondents into groups by considering the frequency with which they consult this information. Subsequently, to check whether there were significant differences in the responses between the different clusters, a Kruskal–Wallis analysis was performed.

Two Chi-square analyses were carried out to evaluate whether there were differences between clusters in the socio-demographic data and in relation to the selection of positive or negative statements related to environmental commitments.

The statistical programmes used for data processing were XLSTAT 2020.3.1 (Addinsoft, Long Island, NY, USA) and Statgpraphics Centurion v.18.1.13 (Statgraphics Technologies Inc., The Plains, VA, USA).

## 3. Results and Discussion

### 3.1. Market Research

During market research, 97 products were identified. Information was collected about brand name (manufacturer’s or distributor’s brands), olive variety, preparation and ingredients, packaging characteristics, and nutrition and/or environmental claims.

This study found a wide variety of table olives on the market, including green Manzanilla olives, black olives (whole and pitted), Cacereña, Kalamata, gordal, aloreña, Amfissa, Chupadedos, Negra de Aragón, Partida, Arbequina, Verdial, and Halkidiki. Of these varieties, Manzanilla green olives dominate the national market with a significant market share. Of all the products, 60% correspond to manufacturer’s brands. Manufacturer’s brands have a wider range of varieties, fillings, dressings, and packaging formats compared to retailer brands. It should be noted that many of the most powerful national companies distribute their products under different brand names. As for the presence of organic labels, 13.4% were identified and belonged to both manufacturers’ and distributors’ brands.

Packaging formats were cans, glass jars, doy-pack pouches, and rigid plastic containers, but cans and glass jars were the most frequent containers. Metal cans are an interesting packaging for manufacturers for their resistance; they can be reused and recycled, and they are a light, airtight, and high-barrier material that protects the product from external agents during storage. Glass jars are the second most usual format on the market for being a high-barrier material that allows the product inside to be seen, like plastic containers. Despite the lightness of doy-pack packaging and the fact that it is easy to transport, distribute, and consume anywhere, it is less frequent on the market because consumers negatively perceive plastic materials and are increasingly aware of the need to reduce plastic waste. Consumers choose to purchase glass or recycled packaging instead of plastic products for their impact on environmental sustainability [14]. This could be related to the large amount of waste caused by food packaging, with up to two-thirds of total packages [15]. Regarding nutritional claims, it was noteworthy that some brands, mainly distributor brands, have a range of low-salt olives identified as follows: “with less salt”, “25% less salt”, “35% less salt”, “70% less salt”, and “low-salt content”. Other labelling claims that might be related to health or wellness were “with omega-3” and “no flavour enhancers”. The presence of these claims highlights the industry’s interest in improving the nutritional profile of its products.

The environmental policies and environmental commitments published on the websites of the main table olive companies were revised. Thirty-five table olive manufacturing companies have either an environmental policy or a section showing the company’s environmental and sustainability commitments, which are publicly accessible. Of these companies, 34.3% have published their environmental policy on their website, which refers to different sections related to sustainability (Table 1).

Of all the environmental commitments, using recyclable materials was the most repeated (19.3%), followed by organic production and zero waste (15.8%). Although most large companies display considerable environmental awareness, their product labelling does not include claims to inform consumers about their commitments, except for organic products, whose labelling indicates that the olives employed as raw material are cultivated by respecting natural systems and cycles. Only the medium and large companies have environmental certifications, such as ISO 14001, EMAS, and ISO 50001 [16], probably because the cost of certification is a limitation for some small companies. As stated in recent studies, although environmentally responsible companies are increasing in number, more than two-thirds of industrial pollution is caused by small- and medium-sized companies, which must adopt eco-friendly operations in their production [17]. Labella et al. reviewed the relevant literature about the ISO 14001 impact on companies’ performance. They detected divergent results due to the differences in country context, company size, and sector [18].

**Figure 2:**
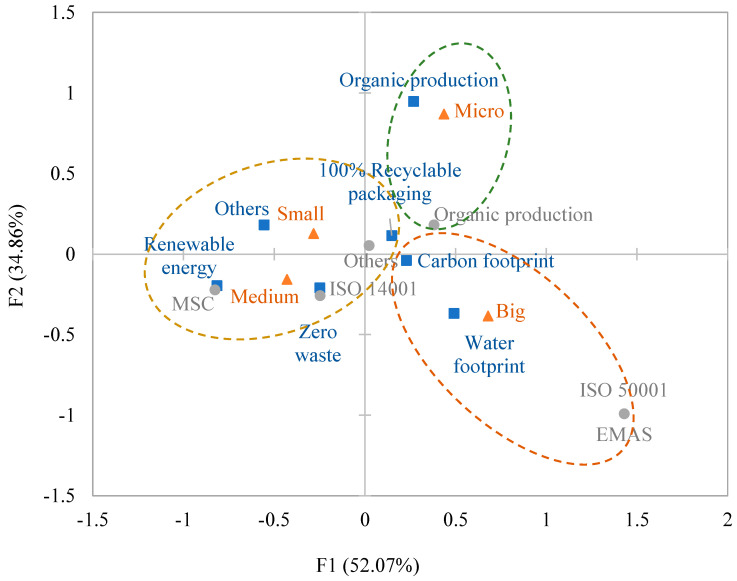
Correspondence analysis for the different environmental commitments and the size of the table olive companies. (▲: Company size; ■: Sustainability commitments; ●: Certifications) [5,16].

To visualise the relations between different commitments and company sizes, a correspondence analysis was carried out (Figure 2). The first two dimensions explained 86.93% of data variance (factor 1, 52.07%; factor 2, 34.86%). “Organic production” was associated with micro-companies. These companies may face sustainable operations because they have a smaller production volume. Big-sized companies make more global commitments, which are correlated with “carbon footprint” and “water footprint”. Small- and medium-sized companies have similar commitments related to “renewable energy”, “zero waste”, and “recyclable packaging”.

### 3.2. Consumer Perceptions Survey

#### 3.2.1. Participants’ Socio-Demographic Data

In this study, 227 consumers participated. More than half were female (59%) and had completed Higher Education (58%). Of the total monthly family income, the highest percentage of respondents (44%) fell within the range from 1500 to 3000 €/month (Table 2).

#### 3.2.2. Consumer Opinion of Table Olive Companies’ Environmental Commitment

In the second questionnaire part, participants marked the options that they associated with each environmental commitment (renewable energy, neutral carbon footprint, lowering the water footprint, zero waste, 100% recyclable packaging, organic products). The frequency of each option for the different environmental commitments is shown in Table 3. Cochran’s Q analysis shows that the selected statements depended on the type of commitment, except for statements 4, 6, and 8, where non-significant differences were observed in commitments.

Respondents selected more positive statements than negative ones for all the sustainability commitments. In this regard, between 85.3% (organic production) and 91.3% (100% recyclable packaging) of the selected options were statements with favourable connotations, compared to 8.6–14.6%, which were negative ones. These results show the importance of companies’ environmental commitments for consumers, who positively value these table olives companies’ environmental actions and policies. These results agree with those obtained by Lami et al. [19], who studied citizens’ commitment to sustainability in consumer habits. They concluded that practically all citizens are aware of the impact that their purchasing actions have on the environment. Moser [20] also evidenced consumers’ growing environmental and sustainable awareness, demanding eco-friendly food, goods, and services. Accordingly, 56% of European consumers claim to be concerned about environmental impacts when buying, and 67% are ready to pay more for eco-friendly products [21]. In particular for table olives, Sánchez-Rodríguez et al. [22] observed that 88% of consumers are willing to pay more than the regular price for a product obtained from olive trees grown in line with water conservation strategies (HydroSOStainable). However, in our study only between 4.1% and 7.8% of consumers stated they were willing to pay more for products from companies with any of the evaluated environmental commitments.

The “100% recyclable packaging” commitment obtained the highest percentage of positive ratings (91.4%), followed by commitments “water footprint” (88.4%) and “zero waste” (88.1%). The fact that participants rated sustainable packaging higher than other commitments could be related to growing consumer concerns about using single-use packaging and its environmental impact. Indeed, European consumers currently pay more attention to the packaging sustainability of the products that they consume, and 90% of consumers would like packaging with extra sustainable information [23]. Similar results have been obtained on other markets outside the EU, where consumers also positively value the use of both eco-friendly packaging and sustainable materials [24]. This substantial change in consumer behaviour regarding packaging has led companies to seek strategies to communicate their own initiatives or actions to reduce the environmental impact of their commercial activity, and this type of statement is increasingly included on food product labelling [25].

The “organic product” commitment received the highest number of negative options (14.65%), such as “It is a hoax” or “It is an excuse to put the price up”. However, it was also the commitment for which more consumers selected option 10 (“I would be willing to pay more for its products”). Although organic production can be considered an added value because consumers believe that organic food is mainly safer and healthier than conventional food [26,27,28], recent studies have shown that some consumers distrust such products [29]. In fact, some consumers are often sceptical about organic claims [30,31,32], mainly due to the diversity of used logos and their doubts about the activity of certifiers and controllers [33,34,35,36]. Options 3 (“It is a company committed to sustainability”) and 9 (“It is a company that respects the environment”) were the least chosen ones for “organic product”, even though, according to EU policies and regulations, organic food production refers to a sustainable agricultural system that respects the environment and animal welfare [37]. Similar results have been observed in previous studies, which have revealed that the reason why consumers choose organic products is mainly for health-related aspects and not for environmental concerns [38]. These results fall in line with those obtained by other researchers, who have concluded that retailers should boost organic food to the environmental- and animal welfare-concerned consumers who are usually willing to pay special prices for these products [39]. However, Dinçer et al. analysed the consumer perception of organic food products in Turkey and revealed two critical issues with organic products, i.e., one is confusion about the organic concept, and the other is the institutional image problem [40].

The “carbon footprint” commitment obtained 84 negative statements (12.3% of the total mentions for this commitment). It should be pointed out that the sector with the largest consumer footprint in Spain is food, which represents 52.1% of the environmental impact [41]. Similarly, Crippa et al. calculated that food production is responsible for 34% of the total greenhouse gas (GHG) emissions [42]. However, consumer awareness about how food production compromises the environment is lacking [43]. Specifically, a Life Cycle Assessment (LCA) of green table olives processing has revealed that the main industrial impact on the environment is GHG emissions from pasteurisation and container production [44]. As the agri-food industry is responsible for one third of the energy globally used [45], consumers should be aware of the responsibility of their actions on the carbon footprint that is generated by a particular product or service [38,46]. Therefore, the food industry must act so that sustainable production systems do not jeopardise the needs of coming generations [47].

It is important to note that only a low percentage of respondents were willing to pay more for the products from companies with these commitments (option 10). The highest percentage corresponded to “organic product” with 7.8% of respondents (52 of the 227 respondents). However, this commitment was also considered “an excuse to put the price up” by the most respondents. This result agrees with those observed by Malissiova et al., who concluded that Greek consumers consider the price of organic food prohibitive for systematic consumption [28]. Option 10 was the least chosen for “renewable energy” with 4.1% of the respondents. Although consumers intend to take a sustainable attitude, it does not have an impact on their purchase decisions [14], and only a few are willing to pay higher prices if the product implies environmental protection and animal welfare [20,39]. Hence, it would be worthwhile to show consumers that environmental initiatives entail making heavy investments and changes in their production models and even substituting their suppliers, which impact product prices [48].

**Figure 3:**
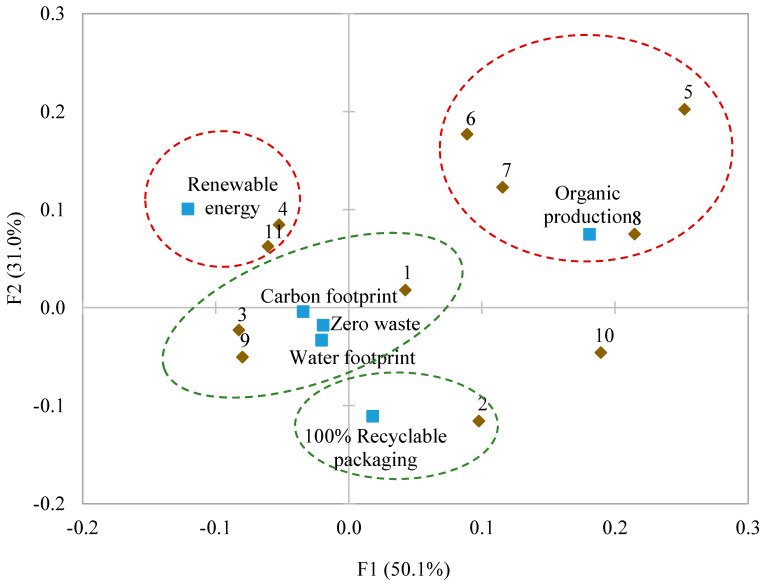
Correspondence analysis for the different environmental commitments and statements (■: Commitments; ◆: Statements). The codes of statements (1–7) are found in Table 3.

To visualise the relations between statements and environmental commitments, a correspondence analysis was carried out. Figure 3 shows the map of the variation in the environmental commitments perception obtained by this analysis. The first two dimensions explained 81.1% of inertia (factor 1, 50.1%; factor 2, 31.0%). “Organic production” was clearly separated from the rest, which was the closest to statements 5–8 and corresponded to the majority of the negative statements (4–8). However, commitments “carbon footprint”, “zero waste”, and “water footprint” occupied an intermediate, closer position to the positive statements. Commitment “100% recyclable packaging” was located the furthest from the negative statements and close to statement 2 (“This company commitment is very important to me”), while the “renewable energy” commitment was separate from the rest and close to statements 4 (“It is only advertising for the brand”) and 11 (“At the same price, I would buy its products”). Statement 10 (“I would be willing to pay more for its products”) was the closest on the map to “organic production” and “100% recyclable packaging”. This reveals that these two commitments would be the most interesting ones for olive producers because this could be reflected in more willingness to pay a premium for their products. This supports previous works that have shown how consumers are willing to pay more for organic food [49,50] or for items packed in sustainable packaging [51]. Traditionally, organic food has been considered healthier than conventional food products [52], and a health concern has been identified as the most important factor when purchasing organic food products. More recent studies have focused on determining consumers’ motivation to purchase organic food by establishing that favourable perceptions of nutritional content and ecological welfare attributes are the main incentives [39,53,54].

When consumers were asked if they consulted food companies’ social and environmental commitments, 42.73% stated that they did not look at this information but were interested in knowing which companies were more committed, while 28.63% of them often checked this information. Around 11–12% of consumers did not check this information because they did not know how to obtain such information or they considered that this information was not useful.

Our findings showed that companies should integrate environmental concerns when developing marketing practices and should also provide information about their environmental commitments and actions to protect the environment.

#### 3.2.3. Checking Packaging Information

In the third questionnaire part, respondents were asked about the frequency with which they reviewed information on packages. The mean values obtained for each attribute are shown in Table 4.

The attributes that respondents most frequently consulted were those related to nutritional aspects, mainly sugars, saturated fats, and calorie content. Information on sugar content was “often” checked by consumers (3.81) and was significantly higher than the rest. This agrees with previous studies, in which consumers stated that they were concerned mainly about overall sugar consumption [55]. These results reveal the importance of nutritional information for most respondents, and it is familiar to consumers because it is mandatory on any food packaging type [56]. In addition, it should be noted that today’s consumers not only buy food to satisfy hunger, but they also seek to obtain an additional benefit for their health. Baudín and Romero [57] determined that 54% of consumers read nutrition labels, of whom 67% admitted consuming products that help them to maintain a healthy diet and 63% analyse the calorie content of foods. Similar results have been found by other studies [58].

The “carbon footprint” parameter obtained the lowest average score (2.13). In general, participants more often checked nutritional information, while environmental information was the least consulted. These results could be related to not only the lack of environmental and sustainability information on packaging but also to the difficulty for consumers to access it, as previously mentioned during market research. In addition, several studies have concluded that environmental protection through eco-labelling is neither trusted by nor understandable for consumers, who do not consider these aspects as part of their daily purchase practices [59,60]. Given growing consumer interest in environmental concerns, the food industry should display easy-to-understand eco-labels that highlight their products’ sustainability.

Three groups were identified in the cluster analysis (Table 4). The individuals in Cluster 1 corresponded to a profile of consumers who do not pay attention to product labelling, with particularly low values for environmental information and animal welfare (close to value 1), “never” in all cases, except for reusable/recyclable packaging (average value equalling 2 “rarely”). Although the values in this cluster for nutritional information were also low, they were higher than the rest of attributes. Finally, Cluster 3 was made up of those respondents who regularly consult any type of information on product labelling. Sánchez-Bravo et al. have studied consumers’ perceptions and attitudes towards food sustainability in different countries and identified three main groups according to their interest in sustainability [61]. In their study, the percentage of Spanish consumers who were very interested in sustainability was significantly higher than in our study. Those authors also observed differences among countries and concluded that consumers in rich countries are more likely to voice more environmental concern.

No significant differences were found among clusters for respondents’ age and family income (Table 2). However, Cluster 1, which was formed by those individuals who never or rarely consulted information on food packaging, was made up of individuals with no education or a low level of education, and this cluster contained more men. On the contrary, the group formed by the most interested consumers in information on labelling (Cluster 3) comprised mainly women and a higher proportion of individuals who had completed university education. There were generally no significant differences among clusters.

To evaluate if there were differences among clusters for the opinion of environmental commitments, a Chi-square analysis was carried out by considering the total positive and negative statements given by respondents about environmental commitments. Although the positive statements predominated in all the groups, the individuals in Cluster 1 (consumers who do not pay attention to product labelling) were those who chose the lowest percentage of positive statements (78%) about environmental commitments. On the contrary, 94.2% of the statements chosen by Cluster 3 (consumers who regularly consult any type of information on product labelling) were positive, and only 5.8% were negative. Cluster 2 was placed between Cluster 1 and Cluster 3. Hence, the consumers who paid more attention to label information were those who trusted the environmental commitments provided by companies. These results agree with D’Souza et al. [62], who stated that the most environmentally concerned consumers are those who usually read product labels.

### 3.3. Limitations and Further Recommendations

The findings of this study have to be seen in light of some limitations. The sample size of consumers is one of the most important limitations because the number of consumers should be bigger to represent the whole Spanish population or the target Spanish market. Moreover, as this study focuses only on the table olive sector, we do not know if the results can be extrapolated to other food industries.

This work can be supplemented with other CATA analyses using different images and claims and by considering other food industries. Conducting more studies in other producer countries would be very interesting for the olive sector. These results could help the food industry to develop the best strategies to publicise its social and environmental policies and commitments based on its target market. Finally, according to the obtained results, it would be interesting to check the effect of consumer formation on environmental concerns.

## 4. Conclusions

The presence of table olives on the Spanish domestic market is substantial, where a wide range of varieties, processing methods, and sales formats can be found and are distributed under manufacturers’ or distributors’ brand names. Although finding claims and/or certifications related to sustainability and the environment in food labelling is becoming increasingly common, these types of indications do not appear on table olive products. However, most of the companies that produce and/or market table olives have adopted environmental and sustainability policies and made commitments, such as lowering the water footprint, zero waste, efficient energy use, and/or taking particular actions against climate change. Consumers positively value these companies’ sustainability commitments and consider that initiatives to use recyclable packaging are very important, while organic products are not especially interesting in the table olives case. Finally, it is necessary to highlight the ambiguity observed for organically produced table olives. Organic products received the fewest positive mentions, but, conversely, consumers stated that they were willing to pay a higher price for them. Seeing that consumers pay close attention to reusable or recyclable packaging, and this is one of the most highly valued environmental commitments, it would be interesting for table olive companies to identify their sustainability commitments on the labelling of their products to, thus, facilitate pro-environmental consumer purchasing choices.

## Figures and Tables

**Table 1:** Characteristics of the table olive companies with environmental and sustainability commitments.

	Number of Companies	%
**Total**	35	100.0
Type		
Olive processing	2	5.7
Packaging	13	37.1
Both	20	57.1
Size		
Big	5	14.3
Medium	14	40.0
Small	10	28.6
Micro	6	17.1
Commitments		
Renewable energy	7	12.3
Carbon footprint	6	10.5
Water footprint	8	14.0
Zero waste	9	15.8
Recyclable packaging	11	19.3
Organic production	9	15.8
Others	7	12.3
Environmental certifications		
Organic production	12	30.0
ISO 14001	13	32.5
ISO 50001	1	2.5
MSC	4	10.0
EMAS	2	5.0
Others	8	20.0
Environmental policy *		
Yes	12	34.3
No	23	65.7

* Environmental policy published on the company’s website.

**Table 2:** Respondents’ data and profile of segments. Frequency (N) and percentage (%) of the total number of respondents.

	Total		Cluster 1		Cluster 2		Cluster 3		χ^2^
	N	%	N	%	N	%	N	%	*p*-Value
	227	100.0	56	24.7	113	49.8	58	25.6	
Gender									
Female	134	59.0	25	44.6	70	61.9	39	67.2	0.0332
Male	93	41.0	31	55.4	43	38.01	19	32.8	
Age									
18–25	46	20.3	13	23.2	22	19.5	11	19.0	0.1938
26–40	90	39.6	19	33.9	47	41.6	24	41.4	
41–50	45	19.8	9	16.1	4	21.2	12	20.7	
51–65	37	16.3	11	19.6	15	13.3	11	19.0	
>65	9	4.0	4	7.1	5	4.4	0	0.0	
Education									
Primary Education	7	3.1	4	7.1	3	2.7	0	0.0	0.0304
Secondary Education	32	14.1	12	21.4	10	8.8	10	17.2	
Higher Education	56	24.7	15	26.8	30	26.5	11	19.0	
University Education	132	58.1	25	44.6	70	61.9	37	63.8	
Monthly family income									
<800 €/month	11	4.9	2	3.6	6	5.3	3	5.2	0.9568
800–1500 €/month	75	33.0	22	39.3	35	31.0	18	31.0	
1500–3000 €/month	100	44.0	23	41.1	50	44.2	27	46.6	
>3000 €/month	26	11.5	6	10.7	15	1.3	5	8.6	
Rather not say	15	6.6	3	5.4	7	6.2	5	8.6	
Relevance of social and environmental commitments									
Yes, I only buy products made by companies committed to the environment	6	2.64	2	3.57	3	2.65	1	1.72	0.8044
Yes, I usually consult the social and environmental commitments of companies	65	28.63	14	25.00	31	27.43	20	34.48	
No. Although I’m interested in knowing which companies are more committed, I don’t look for this information	97	42.73	30	53.57	46	40.71	21	36.21	
No. Although I’m interested, I don’t know how to obtain this information	24	10.57	5	8.93	11	9.73	8	13.79	
No, because I think that most of it is advertising and I don’t feel it is useful	27	11.89	5	8.93	14	12.39	8	13.79	
Others	8	3.52	0	0	8	7.08	0	0	

**Table 3:** The results obtained in the CATA test. Frequency of statement selection (N) and percentage (%) of the total number of mentions of each commitment. *p*-values from Cochran’s Q test. The total number of selected options and the average number of selected statements (M) for each commitment.

Options *	Renewable Energy		Carbon Footprint		Water Footprint		Zero Waste		100% Recyclable Packaging		Organic Production		
	N	%	N	%	N	%	N	%	N	%	N	%	*p*-Value
1	140	20.4	134	19.7	133	19.5	137	20.0	154	20.8	152	22.7	0.002
2	56	8.2	78	11.5	84	12.3	80	11.7	103	13.9	84	12.6	<0.0001
3	139	20.2	135	19.8	136	19.9	138	20.3	140	18.9	103	15.4	<0.0001
4	39	5.7	39	5.7	35	5.1	35	5.1	29	3.9	33	4.9	0.3380
5	9	1.3	7	1.0	5	0.7	10	1.5	8	1.1	15	2.2	0.0360
6	23	3.3	18	2.6	17	2.5	16	2.3	17	2.3	26	3.9	0.0900
7	13	1.9	18	2.6	20	2.9	17	2.5	9	1.2	21	3.1	0.0330
8	1	0.1	2	0.3	2	0.3	4	0.6	1	0.1	3	0.4	0.6760
9	111	16.2	110	16.2	108	15.8	113	16.5	123	16.6	82	12.3	<0.0001
10	28	4.1	37	5.4	39	5.7	36	5.2	49	6.6	52	7.8	<0.0001
11	128	18.6	103	15.1	103	15.1	99	14.4	107	14.5	98	14.6	<0.0001
12	-	-	-	-	-	-	-	-	-	-	-	-	-
	Total	M	Total	M	Total	M	Total	M	Total	M	Total	M	
	687	3.03	681	3.00	682	3.00	686	3.00	740	3.26	669	2.95	

* 1: It is an added value for the product; 2: This company commitment is very important to me; 3: It is a company committed to sustainability; 4: It is only advertising for the brand; 5: It is a hoax; 6: It is an excuse to put the price up; 7: I am indifferent; 8: I prefer companies without these commitments; 9: It is a company that respects the environment; 10: I would be willing to pay more for its products; 11: At the same price, I would buy its products; 12: Other.

**Table 4:** Frequency with which consumers consult environmental, nutritional, and animal production/welfare information on packaging. Values: 1, “Never”; 2, “Very rarely”; 3, “Occasionally”; 4, “Often”; 5, “Always”.

	Total	Cluster 1	Cluster 2	Cluster 3	*p*-Value
**Environmental information**					
Environmentally responsible production system	2.55	1.25 ^a^	2.42 ^b^	4.05 ^c^	<0.0001
Carbon footprint	2.13	1.07 ^a^	1.86 ^b^	3.67 ^c^	<0.0001
Reusable/recyclable packaging	3.37	2.00 ^a^	3.46 ^b^	4.53 ^c^	<0.0001
Reduction in greenhouse gas production	2.42	1.11 ^a^	2.21 ^b^	4.09 ^c^	<0.0001
**Nutritional information**					
Saturated fats	3.63	2.80 ^a^	3.54 ^b^	4.60 ^b^	<0.0001
Calorie content	3.49	2.82 ^a^	3.34 ^a^	4.45 ^b^	<0.0001
Sugars	3.81	3.07 ^a^	3.75 ^b^	4.62 ^b^	<0.0001
Fibre	3.20	1.96 ^a^	3.24 ^b^	4.33 ^b^	<0.0001
**Animal welfare**					
Organic production	2.81	1.52 ^a^	2.76 ^b^	4.17 ^c^	<0.0001
Guaranteed animal welfare	3.00	1.27 ^a^	3.17 ^b^	4.34 ^c^	<0.0001
Animals raised in freedom	3.09	1.63 ^a^	3.12 ^b^	4.43 ^c^	<0.0001
Not tested on animals	3.05	1.41 ^a^	3.16 ^b^	4.41 ^c^	<0.0001

Distinct superscripts indicate significantly different means between clusters for frequency of consultation.

## Data Availability

The original contributions presented in the study are included in the article, further inquiries can be directed to the corresponding author.
